# Is reverse hybrid hip replacement the solution? 3,963 primary hip replacements with cemented cup and uncemented stem, from the Norwegian Arthroplasty Register

**DOI:** 10.3109/17453674.2011.623569

**Published:** 2011-11-25

**Authors:** Einar Lindalen, Leif I Havelin, Lars Nordsletten, Eva Dybvik, Anne M Fenstad, Geir Hallan, Ove Furnes, Øystein Høvik, Stephan M Röhrl

**Affiliations:** ^1^Department of Orthopaedic Surgery, Lovisenberg Deaconal Hospital, Oslo; ^2^Department of Orthopaedic Surgery, Norwegian Arthroplasty Register, Haukeland University Hospital, Bergen; ^3^Section of Orthopaedic Surgery, Department of Surgical Sciences, University of Bergen; ^4^Orthopaedic Department, Oslo University Hospital, University of Oslo, Oslo, Norway

## Abstract

**Background and purpose:**

Reverse hybrid hip replacement uses a cemented all-polyethylene cup and an uncemented stem. Despite increasing use of this method in Scandinavia, there has been very little documentation of results. We have therefore analyzed the results from the Norwegian Arthroplasty Register (NAR), with up to 10 years of follow-up.

**Patients and methods:**

The NAR has been collecting data on total hip replacement (THR) since 1987. Reverse hybrid hip replacements were used mainly from 2000. We extracted data on reverse hybrid THR from this year onward until December 31, 2009, and compared the results with those from cemented implants over the same period. Specific cup/stem combinations involving 100 cases or more were selected. In addition, only combinations that were taken into use in 2005 or earlier were included. 3,963 operations in 3,630 patients were included. We used the Kaplan-Meier method and Cox regression analysis for estimation of prosthesis survival and relative risk of revision. The main endpoint was revision for any cause, but we also performed specific analyses on different reasons for revision.

**Results:**

We found equal survival to that from cemented THR at 5 years (cemented: 97.0% (95% CI: 96.8–97.2); reverse hybrid: 96.7% (96.0–97.4)) and at 7 years (cemented: 96.0% (95.7–96.2); reverse hybrid: 95.6% (94.4–96.7)). Adjusted relative risk of revision of the reverse hybrids was 1.1 (0.9–1.4). In patients under 60 years of age, we found similar survival of the 2 groups at 5 and 7 years, with an adjusted relative risk of revision of reverse hybrids of 0.9 (0.6–1.3) compared to cemented implants.

**Interpretation:**

With a follow-up of up to 10 years, reverse hybrid THRs performed well, and similarly to all-cemented THRs from the same time period. The reverse hybrid method might therefore be an alternative to all-cemented THR. Longer follow-up time is needed to evaluate whether reverse hybrid hip replacement has any advantages over all-cemented THR.

The reverse hybrid method (also known as “inverse hybrid”) uses a cemented all-polyethylene cup in combination with an uncemented stem. This method is partly based on good clinical results of cemented cups and of some uncemented stems in the Norwegian Arthroplasty Register (NAR) (Havelin et al. 2000a,b, Hallan et al. 2007). The register has also shown that some uncemented femoral stems may have better long–term results (> 10 years) than cemented stems in patients 60 years of age or younger. Based on these findings, the NAR suggested 10 years ago that the use of cemented cups in combination with uncemented stems might be justified in young patients (Havelin et al. 2000a). In the Swedish Hip Arthroplasty Register, the performance of uncemented THR was found to be inferior to that of cemented THR (Hailer et al. 2010). The authors of that study found that cemented cups performed better than uncemented cups and that uncemented femoral stems had better survival than cemented stems, with aseptic loosening as endpoint. In the Finnish Arthroplasty Registry, Mäkelä et al. (2010) found better long-term survival regarding aseptic loosening for the best performing types of cementless stems compared to the cemented reference group, in the age group 55–74 years.

McNally et al. (2000) studied survival of the Furlong HA coated femoral stem in combination with a cemented ultra-high-density polyethylene cup at 10–11 years, and found values of 99% for the stem and 95% for the cup. Alho et al. (2000) reported results with cemented Lubinus cups and uncemented Furlong stems, and they also pointed out the possibility of using the principle of reverse hybrid arthroplasty. We are not aware of any other reports on the reverse hybrid method.

In a reverse hybrid THR, an uncemented stem and a modular head are most often combined with a cemented cup of another name or from another company. Combining implants that are not designed to fit each other might theoretically lead to unexpected complications such as increased wear, loosening, or dislocation. This concern was raised by the NAR already in their report from 2005 (Norwegian Arthroplasty Register 2005). As the use of reverse hybrids is increasing, we decided to evaluate the short- to medium-term results with this concept and to compare them with those from all-cemented THRs, using data from the NAR.

## Patients and methods

The NAR was established in September 1987 (Havelin et al. 1993). Data on primary and revision THR surgery are collected, and the patients are followed prospectively until revision, death, or emigration. The unique identification number assigned to each resident of Norway makes it possible to link the primary operation to revision surgery and to the National Population Register, which provides information on death or emigration. Completeness of registration is high for total hip replacement, for both primary and revision surgery (Arthursson et al. 2005, Espehaug et al. 2006, Hulleberg et al. 2008).

**Figure 1.**
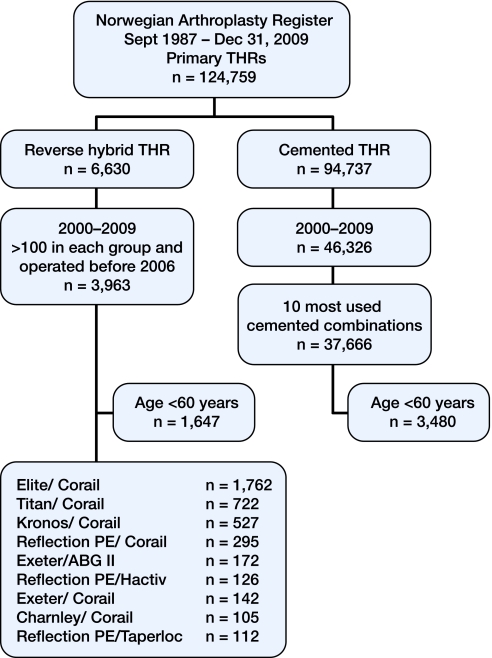
Flowchart of the study.

From September 1, 1987 through December 31, 2009, 124,759 primary THRs were registered. Of these, 6,630 cases involved reverse hybrid THR. 15 different cups and 13 different femoral stems had been used for these reverse hybrids. Since reverse hybrid THR has mainly been used during the last decade, we included only operations performed after December 31, 1999. This gave 6,485 primary operations. We included only the combinations of cup and stem for which there had been more than 100 procedures since 2005. Thus, 3,963 operations in 3,630 patients were included (Figure 1) for survival estimation at 5 and 7 years, involving 9 implant combinations (cup/stem) (Tables 1 and 2). In these implant combinations, all cups were made from conventional ultra-high-molecular-weight polyethylene (UHMWPE).

**Table 1.** Breakdown of numbers of different combinations of prosthesis components in the reverse hybrid group during the study period

Combination of cup and stem (reverse hybrid)										
Year	Elite/Corail	Titan/Corail	Kronos/Corail	Reflection PE**^a^**/Corail	Exeter/Corail	Charnley/Corail	Exeter/ABG II	Reflection PE**^a^**/Hactiv	Reflection PE**^a^**/Taperloc	Total
2000	38	3	3	6	1	6	0	0	0	57
2001	43	22	14	13	2	4	0	0	0	98
2002	45	46	6	37	3	7	0	0	0	144
2003	61	50	9	55	8	2	61	0	0	246
2004	83	72	14	63	16	9	50	12	0	319
2005	178	79	39	69	10	5	61	37	34	512
2006	269	82	66	18	11	14	0	29	49	538
2007	365	75	98	26	18	34	0	18	29	663
2008	362	113	157	5	28	13	0	16	0	694
2009	318	180	121	3	45	11	0	14	0	692
Total	1762	722	527	295	142	105	172	126	112	3963

**^a^** Full brand name: Reflection Cemented All-Poly.

**Table 2.** Comparison of different combinations of prosthesis components in the reverse hybrid group

Brand name cup/stem	Manufacturer cup/stem	N	Revisions	Median follow-up (range)	Mean age (min–max)	% < 60 year	% male	No. of hospitals (max % at hospital)
Elite/Corail	Landos Depuy/Depuy	1762	40	2.6 (0–10)	60 (21–92)	48	40	15 (34%)
Titan/Corail	Landos Depuy/Depuy	722	21	3.0 (0–9.8)	63 (27–91)	36	36	11 (34%)
Kronos/Corail	Landos Depuy/Depuy	527	12	1.8 (0–9.7)	63 (20–92)	33	32	4 (83%)
RPE **^a^**/Corail	Smith & Nephew/Depuy	295	17	5.6 (0–9.9)	58 (18–90)	58	37	11 (35%)
Exeter/ ABG II	Stryker **^b^**/Stryker	172	6	5.4 (1.4–7.0)	73 (50–88)	2	37	2 (82%)
RPE **^a^**/Hactiv	Smith & Nephew/Scanos **^c^**	126	5	3.6 (0–5.6)	64 (19–91)	38	37	2 (97%)
Exeter/Corail	Stryker **^b^**/Depuy	142	2	1.9 (0–9.9)	64 (19–87)	35	18	5 (67%)
Charnley/Corail	Depuy/Depuy	105	2	2.8 (0.4–9.9)	58 (21–86)	61	21	14 (46%)
RPE **^a^**/Taperloc	Smith & Nephew/Biomet	112	3	3.8 (2.5–4.7)	61 (40–82)	36	33	1 (100%)

**^a^** Full brand name: Reflection Cemented All-Poly.

**^b^** Full brand name: Stryker, Osteonics, Howmedica

**^c^** Full brand name: Scanos Evolutis

From the register, we extracted information on the brand(s) of the components, the diameter and the material of the femoral heads, the diagnosis, the name of the hospital, the surgical approach to the hip, and reasons for revision surgery. We estimated survival at 3, 5, and 7 years for the total material with any revision as the endpoint. Further subgroup analyses included survival at 3, 5, and 7 years in patients less than 60 years of age, with any revision as endpoint. Furthermore, we compared reverse hybrid THR to cemented THR for the total material, with deep infection, dislocation, aseptically loosened stem, and aseptically loosened cup as endpoint in the same period.

We compared the results to the 10 most commonly used cemented cup/stem combinations in the study period. These cemented implants and the cups in the reverse hybrid group have been described by Espehaug et al. (2009) (Table 3). Details of the stems in the present study are given in Table 4. We excluded patients operated with CMW cement, due to the poor results described by others after use of this cement (Havelin et al.1995, Espehaug et al. 2002).

**Table 3.** Cup/stem combinations in the cemented group. These have been thoroughly described by Espehaug et al. (2009)

	Manufacturer	Number of prostheses
Charnley/Charnley	Depuy	12,192
Exeter/Exeter	Stryker, Osteonics, Howmedica	6,419
Reflection PE/Spectron	Smith & Nephew	8,618
Titan/Titan	Landos, Depuy	2,736
Spectron/ITH	Smith & Nephew	162
Link IP/Lubinus SP(I,II)	Waldemar Link	2,203
Contemporary/Exeter	Stryker, Osteonics, Howmedica	2,707
Kronos/Titan	Landos, Depuy	1,073
Elite/Titan	Depuy/Landos Depuy	1,139
Reflection/ITH	Smith & Nephew	417

**Table 4.** Details of the characteristics of the uncemented femoral stems used in the reverse hybrid group. 97% had HA coating

Stem	Material	Shape	Surface	Thickness of HA	Company
Corail	Ti6A14V	Straight, tapered	Fully HA-coated	155 µm	DePuy
ABG II	Ti alloy	Anatomic	HA-coated proximal, polished distally	50 µm	Stryker
Hactiv	Ti6A14V	Straight, tapered	Fully HA-coated	155 µm	Evolutis
Taperloc	Ti6A14V	Straight, tapered	Without HA in this study, proximal plasma spray coating		Biomet

### Statistics

Risk Ratio (RR) with 95% confidence interval (CI) was estimated using Cox regression analyses, with adjustments for age (< 50, 50–59, 60–69, 70–79 and > 80), sex, and diagnosis (osteoarthritis (OA), inflammatory arthritis, and others). We used plots with scaled Schoenfeld residuals for each covariate to test that the Cox proportional hazard model was fulfilled. The Kaplan-Meier method was used for estimation of survival probabilities for the prostheses, with 95% confidence interval (CI). Ranstam and Robertsson (2010) have discussed statistical analysis regarding arthroplasty register data and found a negligible effect on survival estimates including bilateral hips. We therefore included bilateral hips. When less than 20 hips remained at risk, survival probabilities were not calculated. Median follow-up was calculated using the reverse Kaplan-Meier method. We used chi-squared test to test for binary outcomes between study groups, and the non-parametric Mann-Whitney test was used to determine whether the distribution of medians was different between study groups. All p-values less than 0.05 were considered to be statistically significant. We used the statistical software packages SPSS (SPSS 17.0 for Windows) and R (version 2.8.1; http://www.R-project.org).

## Results

The mean age was lower in the reverse hybrid group than in the cemented group: 61 (18–92) years as opposed to 73 (16–98) years. The proportion of males was higher in the reverse hybrid group than in the cemented group (36% vs. 29%). In addition, 9% of patients were below 60 years of age in the cemented group and the corresponding proportion in the reverse hybrid group was 42%. Furthermore, there were significant differences regarding diagnosis, age, and sex (Table 5). In the total material, median follow-up was 2.9 (0–10) years in the reverse hybrid group and 4.7 (0–10) years in the cemented group. For patients aged less than 60 years, the median follow-up was 3.4 (0–10) years for reverse hybrid and 5.2 (0–10) years for cemented (Table 5).

**Table 5.** Comparison of demographic data for cemented and reverse hybrid THRs, both for total material and for patients aged < 60 years. Comparison of survival (in %) and relative risk (RR) of revision for cemented and reverse hybrid THRs, with all revisions as endpoint, for total material and for patients aged < 60 years

	Total material			Age < 60 years		
	Cemented	Reverse hybrid	p-value	Cemented	Reverse hybrid	p-value
n	37,666	3,963		3,480	1,647	
Revisions	1,140	108		135	41	
Median follow-up (range)	4.7 (0–10)	2.9 (0–10)	< 0.001 **^b^**	5.2 (0–10)	3.4 (0–10)	< 0.001 **^b^**
Mean age (min–max)	73 (16–98)	61 (18–92)	< 0.001 **^b^**	54 (16–60)	52 (18–60)	< 0.001 **^b^**
% < 60 years	9	42	< 0.001 **^a^**	100	100	
% male	29	36	< 0.001 **^a^**	36	39	0.06 **^a^**
Deceased	5,928 (15.7%)	104 (2.6%)		229 (6.6%)	25 (1.5%)	
Emigrated	58 (0.2%)	8 (0.2%)		21 (0.6%)	4 (0.4%)	
Missing	2	0		0	0	
Alive	31,678 (84.1%)	3,851 (97.2%)		3,230 (92.8%)	1,618 (98.2%)	
Diagnosis			< 0.001 **^a^**			< 0.001 **^a^**
Osteoarthritis	78.6%	70.9%		56.6%	55.9%	
RA/Inflammatory	3.3%	4.2%		8.0%	6.0%	
Sequelae hip fracture	8.9%	5.2%		7.1%	4.8%	
Dysplasia	4.3%	11.6%		16.5%	20.4%	
Perthes'	0.6%	2.2%		3.1%	4.5%	
Other	4.4%	5.9%		8.7%	8.4%	
3-year survival (95%CI)	97.9 (97.7–98.0)	97.7 (97.2–98.2)		98.0 (97.5–98.5)	98.3 (97.7–99.0)	
5-year survival (95%CI)	97.0 (96.8–97.2)	96.7 (96.0–97.4)		96.7 (96.0–97.3)	97.5 (96.6–98.5)	
7-year survival (95%CI)	96.0 (95.7–96.2)	95.6 (94.4–96.7)		94.9 (94.0–95.9)	96.2 (94.6–97.8)	
RR **^c^** (95%CI)	1 (Reference)	1.1 (0.9–1.4)	0.3	1 (Reference)	0.9 (0.6–1.3)	0.5

**^a^** Chi-squared test.

**^b^** Non-parametric Mann-Whitney.

**^c^** RR adjusted for age, sex, and diagnosis.

**Figure 2.**
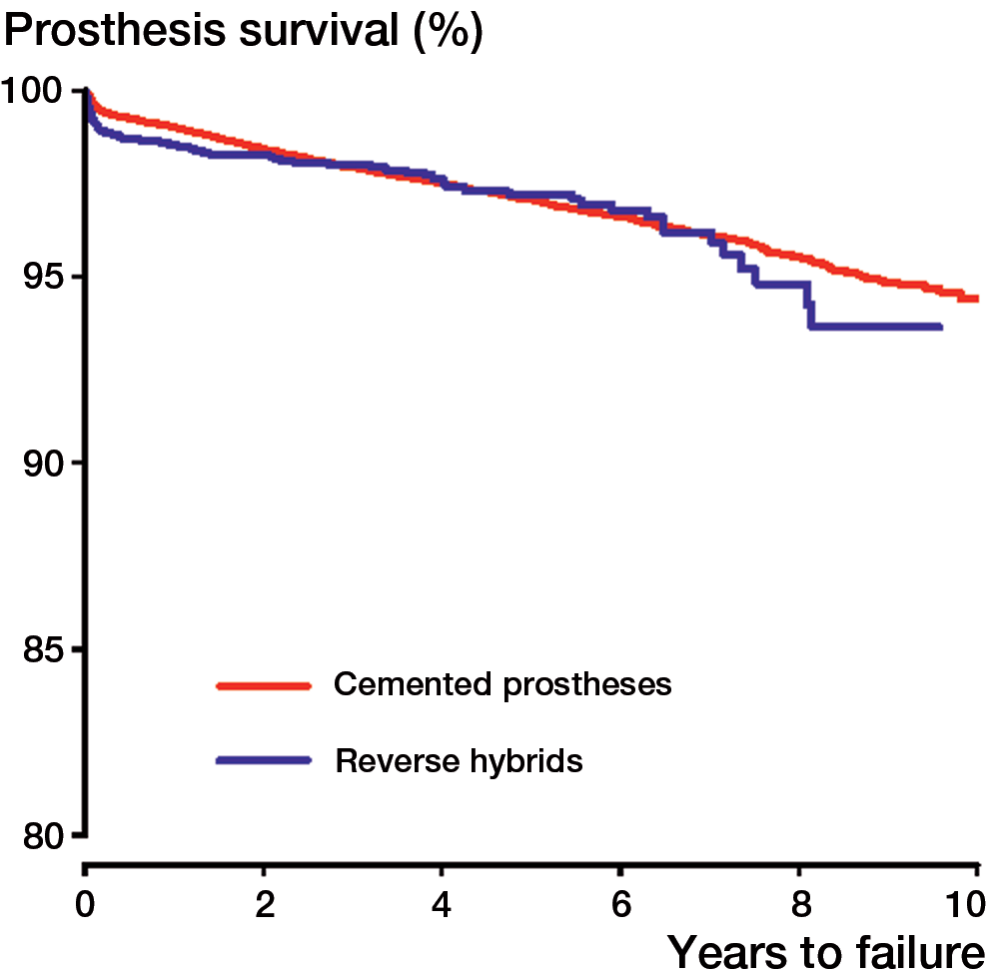
Cox survival curves. Endpoint was any revision of the implant for the total material. Adjusted for age, sex, and diagnosis. Age: < 50, 50–59, 60–69, 70–79 and > 80. Diagnosis: OA, RA/inflammatory, or other.

**Figure 3.**
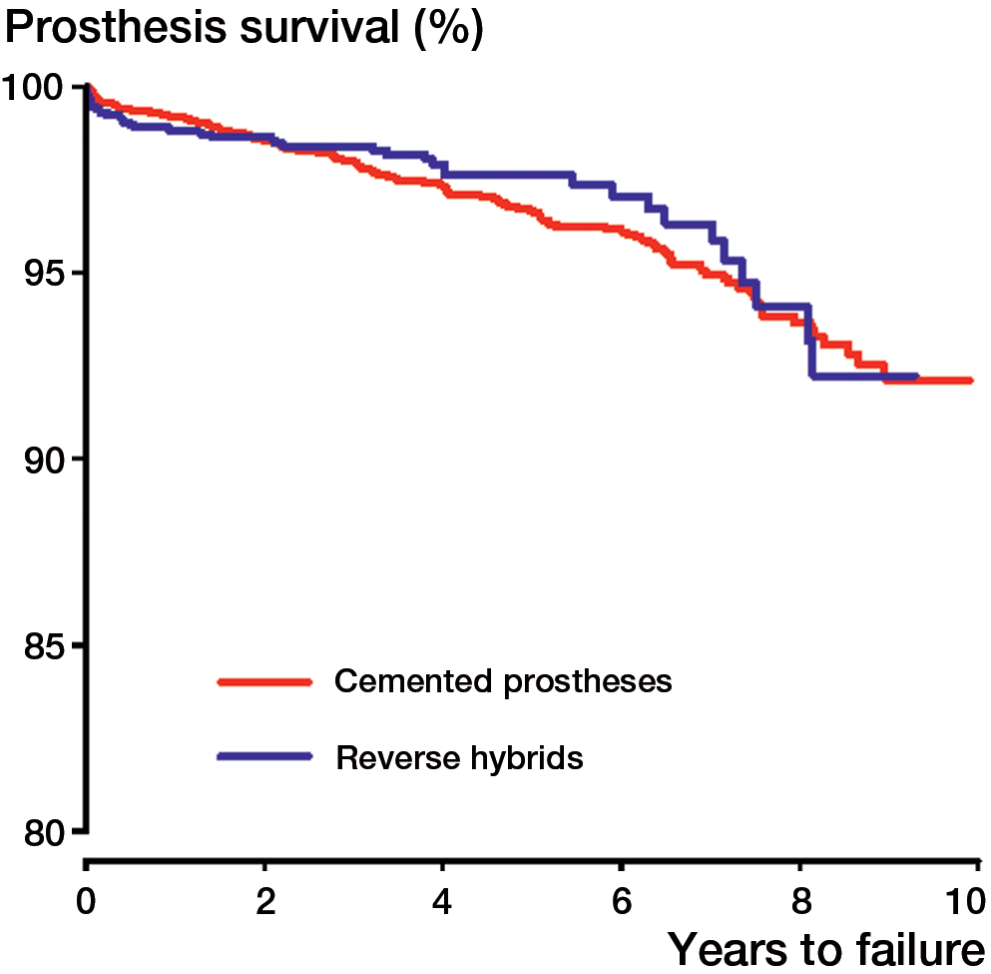
Cox survival curves. Endpoint was any revision of the implant. Age < 60 years. Adjustment for age, sex, and diagnosis. Age: <50, 50–60. Diagnosis: OA, RA/inflammatory, or other.

There was no statistically significant difference in implant survival between cemented and reverse hybrid THRs when the endpoint was any revision. This was also found in analyses of cases less than 60 years of age (Figures 2 and 3, Table 5). In subanalyses of the total material using the endpoints revision due to deep infection, dislocation, aseptically loosened stem, and aseptically loosened cup, no statistically significant differences between cemented and reverse hybrid THRs were found.

The reverse hybrids had a 3.6 times higher risk of revision for periprosthetic femoral fracture compared to the cemented implants (CI: 1.9–6.9; p < 0.001). The survival was 99.85% (99.8–99.9) and 99.6% (99.3–99.9), respectively, at 5 years using this endpoint.

We performed analyses of the different cup/stem combinations in the reverse hybrid group with all revisions as the endpoint at 3 and 5 years. No statistically significant differences were found. Reflection PE/Hactiv and Reflection PE/Taperloc had less than 20 hips left at risk at 5 years; thus, 5-year survival of these combinations could not be estimated.

In the reverse hybrid group, 3,832 of the 3,963 prostheses had a femoral head with a diameter of 28 mm. 2,467 heads were made of alumina and 1,286 heads were made of cobalt chromium. In these 2 groups, all head sizes were included.

Among the different groups of reverse hybrid combinations, we noted differences in age, in median follow–up time, and in the male/female ratio. The ReflectionPE/Taperloc combination has been used since 2005, but maximum follow-up for this group only reached 4.7 years. All the other groups of reverse hybrid combinations had a maximum follow-up of more than 5 years (Table 2).

## Discussion

The use of reverse hybrids has increased in Norway and Sweden during the last decade. Before 2000, few reverse hybrid operations were performed each year, and with many different combinations of components. In this pre-2000 period, we believe that in some instances failure to achieve solid fixation of an uncemented cup made the surgeon convert to a cemented cup. Since the year 2000, the reverse hybrid concept has been used more systematically in Norway and the number of implanted primary reverse hybrids has increased from 90 in the year 2000 to 1,735 in 2009. In Sweden, the number of hybrid THRs has declined and the total number of reverse hybrids has increased (Swedish Hip Arthroplasty Register 2007). This increase in popularity called for the evaluation of medium-term results using this method.

Comparing the reverse hybrid group with the 10 most used cemented THRs, we found similar implant survival with 0–10 years of follow-up. The only differences found were for subanalyses on femoral fractures, but the difference in survival at 5 years with this endpoint was only 0.25% and 5-year survival exceeded 99% for both groups. This indicates that periprosthetic femoral fractures are an infrequent complication leading to revision surgery.

For the total material, the proportion of males was higher and the mean age was lower in the reverse hybrid group than in the cemented group. We had no scoring for activity level, and there could be a bias in comparing high-demand young men to a group with low-demand elderly women. However, when we limited analyses to patients less than 60 years of age, the groups were much more similar to each other. The median follow-up for total material and for cases below 60 years differed significantly between study groups, and with short follow-up it may therefore be difficult to uncover differences between these 2 concepts.

We found similar risk of deep infection with reverse hybrid and cemented THR. Only revisions that included removal or exchange of parts or the whole implant were reported to the register. Thus, soft tissue revisions without the exchange of prosthetic parts were not reported to the NAR. For the period 2003–2007 and using data from the NAR, Dale et al. (2009) found a statistically significant difference in numbers of revisions due to deep infection with inferior results for uncemented THR compared to cemented THR. One explanation for our finding is that antibiotic in the cement in reverse hybrids may protect against deep infection (Engesaeter et al. 2003).

In the present study, 97% of the stems had HA coating and in the medium term we found results comparable to those for cemented THR (Table 4). In 2002, the NAR reported inferior results for 2 types of HA-coated cups as compared to cemented Charnley cups (Havelin et al. 2002). In 2010, Lazarinis et al. reported increased risk of revision of acetabular cups coated with HA, and in 2009 Stilling et al. reported inferior results for an HA-coated cup compared to those for a non HA-coated cup at 15 years. Concerns have been raised about third body wear induced by HA from HA-coated implants. Røkkum et al. (2002) discussed whether thick HA coatings may delaminate, and suggested that thick HA coatings may be a reservoir for HA particles. Wear and wear-related problems may appear several years after the primary procedure. Studies with large numbers and long follow-up are thus necessary in order to be able to conclude whether the performance of cup implants is influenced by the stem having an HA coating. Regarding this problem, randomized controlled trials measuring wear with precise methods are important, but registry studies collecting a large amount of data on prostheses may also reveal differences between HA-coated implants and those without any HA coating.

In the NAR, femoral fractures are reported if they require revision surgery. We found a higher risk of revision for periprosthetic femoral fracture in the reverse hybrids than in the all-cemented THRs. Although it was more common with uncemented stems, periprosthetic femoral fracture was uncommon in both groups. Hailer et al. (2010) found in a study from the Swedish Arthroplasty Register that uncemented stems were more frequently revised due to periprosthetic fracture than cemented stems during the first 2 postoperative years.

We used the Kaplan-Meier method to estimate prosthesis survival, censoring death and emigration. Both death and emigration are competing risks regarding revision. In a study from the Australian Orthopaedic Association National Joint Replacement Registry, Gillam et al. (2010) found that the Kaplan-Meier method overestimated the risk of revision compared to a method called the cumulative incidence function. The latter method uses competing risk methods in the analyses. With a short- to medium-term follow-up and a rather low incidence of death, we assumed that the Kaplan-Meier method would be appropriate to use in this study.

Regarding revision due to deep infection, dislocation, aseptically loosened stem, and aseptically loosened cup, we did not find any statistically significant difference between cemented and reverse hybrid THRs. In planning the study, we aimed to do subanalyses with the endpoints revision due to deep infection, dislocation, aseptically loosened stem, and aseptically loosened cup for the different combinations of cup/stem (different brands) in the reverse hybrid group. We found that the number of revisions and the number of procedures in some groups were quite small (Table 2). Thus, 1 single revision would have a large effect on the survival calculations for certain implant combinations. Although our register has a high completeness of data, we do not know for certain that all revisions of the primary THRs included were reported to the register. 1 or 2 missing revisions in 1 study group may offset the results quite dramatically when the groups are small. Furthermore, the accuracy of registry results is not known; the surgeon may type the data into the wrong box on the form, or the register may enter wrong data into the database. It is therefore difficult to make conclusions about the performance of the different components used in the reverse hybrid group. Subtle differences between study groups, if found, should be interpreted with caution—even if they are statistically significant. Factors other than the implant itself, such as surgical technique, revision policy, incorrect registration, or unknown patient factors may bias the results.

In summary, we found no statistically significant differences in survival between reverse hybrid and all-cemented THRs in this population-based registry study. Both groups performed well, with 95–96% survival after up to 7 years of follow-up. Thus, there were no early signs of warning against the reverse hybrid method according to our findings. Due to the small number of revisions in the present study, we cannot make any conclusions regarding the results for the different cup/stem combinations of reverse hybrid THR. With a short- to medium-term follow–up, it appears that the reverse hybrid method might be a promising alternative in THR surgery using UHMWPE. We emphasize that long-term follow-up will be required to evaluate whether the concept has any advantage over all-cemented THR.
